# The lysosomal enzyme receptor protein (LERP) is not essential, but is implicated in lysosomal function in *Drosophila melanogaster*

**DOI:** 10.1242/bio.013334

**Published:** 2015-09-24

**Authors:** Medina Hasanagic, Eline van Meel, Shan Luan, Rajeev Aurora, Stuart Kornfeld, Joel C. Eissenberg

**Affiliations:** 1 Edward A. Doisy Department of Biochemistry and Molecular Biology, Saint Louis University School of Medicine, St. Louis, MO 63104, USA; 2 Department of Internal Medicine, Washington University School of Medicine, Saint Louis, MO 63110, USA; 3 Department of Biology, Saint Louis University, St. Louis, MO 63103, USA; 4 Department of Molecular Microbiology and Immunology, Saint Louis University School of Medicine, St. Louis, MO 63104, USA

**Keywords:** Lysosomal enzyme receptor protein, Lysosomal sorting, *Drosophila* sorting receptor

## Abstract

The lysosomal enzyme receptor protein (LERP) of *Drosophila melanogaster* is the ortholog of the mammalian cation-independent mannose 6-phosphate (Man 6-P) receptor, which mediates trafficking of newly synthesized lysosomal acid hydrolases to lysosomes. However, flies lack the enzymes necessary to make the Man 6-P mark, and the amino acids implicated in Man 6-P binding by the mammalian receptor are not conserved in LERP. Thus, the function of LERP in sorting of lysosomal enzymes to lysosomes in *Drosophila* is unclear. Here, we analyze the consequence of LERP depletion in S2 cells and intact flies. RNAi-mediated knockdown of LERP in S2 cells had little or no effect on the cellular content or secretion of several lysosomal hydrolases. We generated a novel *Lerp* null mutation, *Lerp^F6^*, which abolishes LERP protein expression. *Lerp* mutants have normal viability and fertility and display no overt phenotypes other than reduced body weight. *Lerp* mutant flies exhibit a 30–40% decrease in the level of several lysosomal hydrolases, and are hypersensitive to dietary chloroquine and starvation, consistent with impaired lysosome function. Loss of LERP also enhances an eye phenotype associated with defective autophagy. Our findings implicate *Lerp* in lysosome function and autophagy.

## INTRODUCTION

In mammalian cells, the two mannose 6-phosphate (Man 6-P) receptors (MPRs), cation-independent (CI) and cation-dependent (CD) MPRs, function to transport newly synthesized lysosomal acid hydrolases from the trans-Golgi network (TGN) to the endosomal/lysosomal system (Ghosh et al., 2003). These receptors bind the acid hydrolases via Man 6-P tags that are added to the hydrolases in the cis*-*Golgi and simultaneously bind adaptor proteins, GGAs and AP-1, for their incorporation into clathrin-coated vesicles at the trans-Golgi interface. Interestingly, Dennes et al. identified a single MPR ortholog in *Drosophila melanogaster* that was termed LERP, for lysosomal enzyme receptor protein (Dennes et al., 2005). LERP is a type I transmembrane protein whose lumenal domain contains five repeats that share overall homology with the 15 lumenal repeats of the CI-MPR. LERP is localized to the TGN and endosomes in *Drosophila* S2 cells and interacts with the adaptor proteins GGA and AP-1 via acidic dileucine and tyrosine-based sequences in its cytoplasmic tail (Hirst et al., 2009; Kametaka et al., 2010). Furthermore, LERP is incorporated into clathrin-coated vesicles by a process that is dependent on GGA and AP-1 (Hirst et al., 2009). These features are consistent with LERP functioning as a receptor involved in transporting cargo from the TGN to its destination. In support of this concept, Dennes et al. expressed LERP in MPR-deficient mouse fibroblasts and reported that it partially rescues the missorting of several lysosomal acid hydrolases (Dennes et al., 2005). However, these investigators found that LERP fails to bind to a phosphomannan affinity column, and the amino acids implicated in Man 6-P binding in mammalian MPRs are not conserved in LERP. Additionally, the *Drosophila* genome lacks discernable homologs for genes encoding essential enzymes for the Man 6-P mark, the gamma subunits of the N-acetylglucosamine-1-phosphate transferase and the N-acetylglucosamine-1-phosphodiester alpha-N-acetylglucosaminidase uncovering enzyme. This suggests that the Man 6-P-dependent sorting mechanism is absent in flies. Most recently, Kowalewski-Nimmerfall et al. reported that RNAi knockdown of LERP in S2 cells had only a small effect on the retention of the lysosomal enzyme cathepsin L and no effect on lysosomal CREG (cellular repressor of EIA-stimulated genes retention), leading them to suggest that LERP is not a universal sorting receptor for lysosomal proteins in flies (Kowalewski-Nimmerfall et al., 2014).

To clarify these paradoxical results and to test the role of LERP in the whole fly, we generated a *Lerp* null *Drosophila* mutant and investigated the impact on development and on lysosomal enzyme sorting and lysosome-dependent phenotypes. We also analyzed the consequence of LERP knockdown in S2 cells on the trafficking of several lysosomal hydrolases.

## RESULTS

### Depletion of LERP in *Drosophila melanogaster* S2 cells

**Fig. 1.**
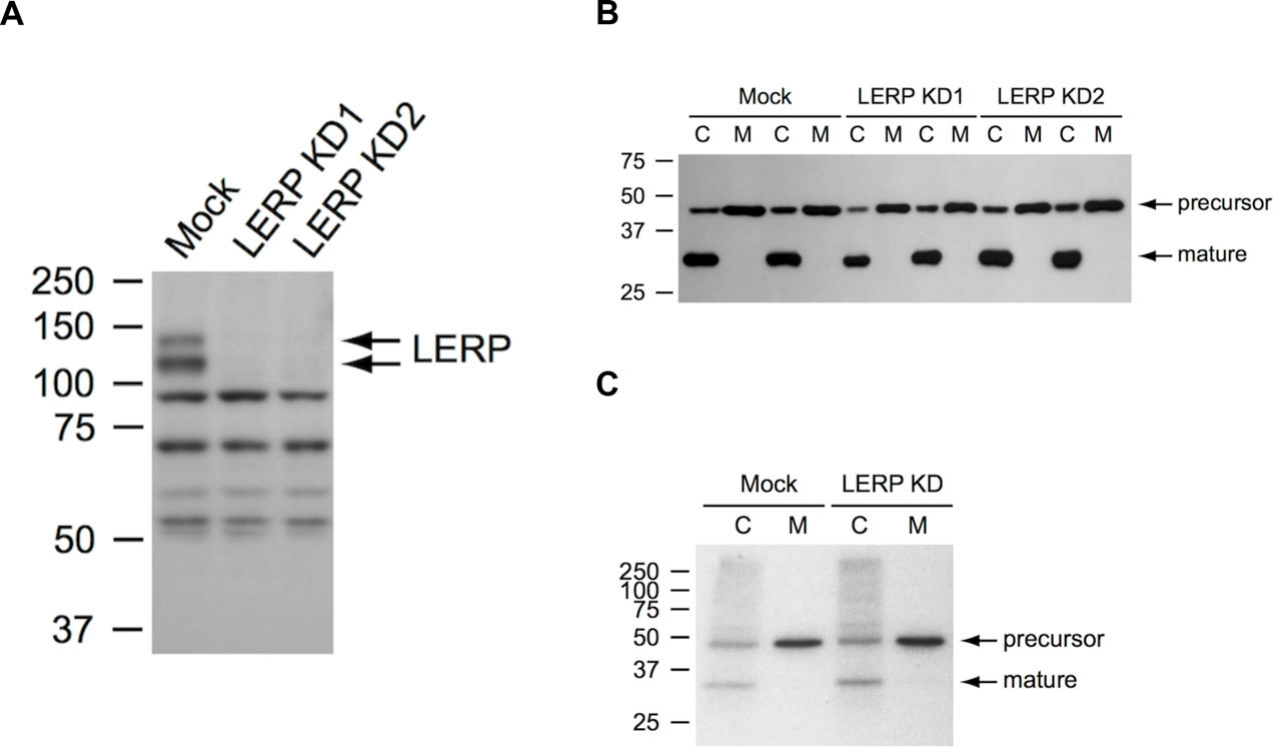
**Cathepsin L sorting in LERP-depleted *Drosophila* S2 cells.** (A) Western blot showing LERP depletion in RNAi-treated S2 cells (5 days). Equal amounts of total protein were loaded, with 25 μg of protein/lane. (B) Western blot of cathepsin L in S2 cell lysates (C) and media (M) after 9 days of knockdown. For each lysate, 15 μg protein was loaded and 2 times the volume of medium. No significant differences were observed in the ratio of precursor to mature cathepsin L in LERP depleted versus mock-treated cells. (C) Pulse-chase labeling and cathepsin L immunoprecipitation in S2 cells. C, cell lysates; M, media. Similar levels of the precursor and mature form of cathepsin L were present in the mock-treated and LERP depleted cell lysates. In both instances, 49% was secreted into the culture medium.

To explore the possibility that LERP functions as a sorting receptor for lysosomal enzymes at the TGN, the consequence of LERP depletion was first studied in *Drosophila* S2 cells using RNAi-mediated knockdown. In these experiments, we would predict that loss of LERP would impair the lysosomal targeting of these enzymes. Additionally, it would lead to reduced intracellular levels of lysosomal enzymes due to enhanced cellular secretion via the constitutive secretory pathway. The S2 cells were treated with LERP dsRNA for five days, with fresh media added 16 h prior to harvesting the cells. Cell lysates were then prepared and aliquots of these lysates and media were assayed for their content of a panel of lysosomal glycosidases (Table 1). The mock-treated cells showed various degrees of glycosidase secretion over the 16 h collection period, ranging from 12% of total β-hexosaminidase to 95% of β-galactosidase. With the exception of a 19% increase in the secretion of β-glucuronidase, LERP depletion had no effect on the secretion of the other glycosidases tested relative to mock treated cells. Furthermore, the cellular content of these glycosidases was unchanged relative to mock treated cells, aside from a small decrease in cellular β-glucuronidase. The knockdown of LERP mRNA was >88% as determined by RT-PCR, while the depletion of LERP protein was confirmed by western blotting (Fig. 1A). Similar results were obtained with prolonged knockdown of nine days; the cellular content of β-glucuronidase was not decreased relative to the mock-treated cells (data not shown).

**Table 1.**
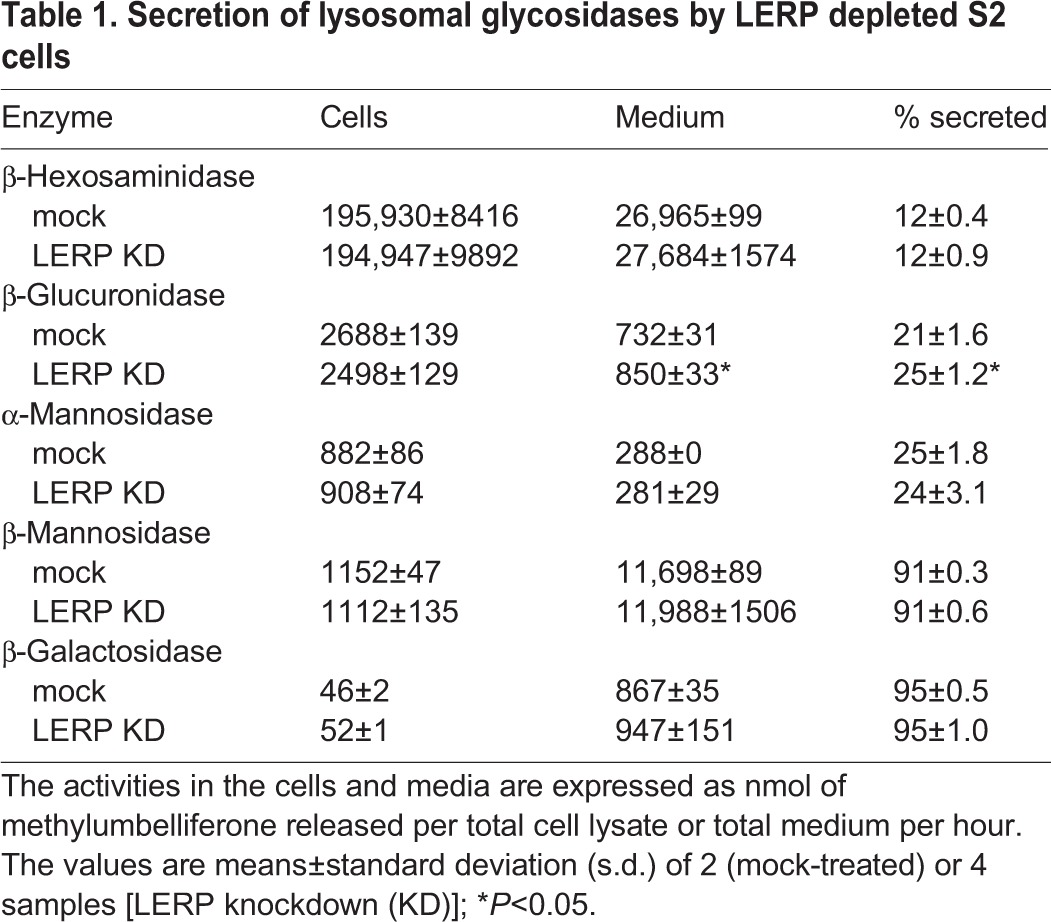
**Secretion of lysosomal glycosidases by LERP depleted S2 cells**

In another experiment, the levels of cathepsin L, a lysosomal endopeptidase, were determined by western blotting. In both mock-treated and LERP depleted cells, the cathepsin L precursor (∼45 kDa, inactive pre-lysosomal) and mature (∼30 kDa, lysosomal) forms were detected in the cell lysates (Fig. 1B). In media samples, however, only the precursor of cathepsin L was detected. Impaired lysosomal targeting of cathepsin L would shift the ratio of precursor to mature enzyme in the cells towards the precursor form and in addition, increase the precursor levels in the media. However, no differences in cathepsin L sorting were observed after five or nine days of LERP depletion compared to mock-treated cells (Fig. 1B). To quantify the effect of LERP depletion on cathepsin L sorting, pulse-chase labeling experiments were performed. In both mock-treated and LERP depleted S2 cells, 49% of cathepsin L was secreted into the culture medium (Fig. 1C). Taken together, these results are not consistent with a role for LERP as a universal receptor for lysosomal enzymes.

Utilizing affinity chromatography, we attempted to directly test whether LERP binds lysosomal enzymes. A soluble form of LERP, expressed in *Spodoptera frugiperda* (Sf9) cells, was immobilized on an affinity resin and S2 cell lysates or medium were passed over the column as a source of *Drosophila* lysosomal enzymes. Using this system, we did not observe any binding of lysosomal enzymes, including β-hexosaminidase, β-glucuronidase, α-mannosidase, and β-mannosidase, to the immobilized LERP (data not shown). Because these studies regarding the role of LERP in *Drosophila* S2 cells were inconclusive, we focused on the intact organism.

### Generation and characterization of a *Lerp* null allele

Three strategies were attempted in order to generate a *Lerp* knockout mutation. The first two relied on mobilizing excisions of existing P-element transposon insertions at the *Lerp* locus to generate local deletions within *Lerp*. In one strategy, the *Mi{ET1}Lerp^MB05321^* insert near the 3′ end of *Lerp* was mobilized by crosses to a stock carrying the *HoP2.1* transposase transgene. Among the 276 progeny showing loss of the *Mi{ET1}Lerp^MB05321^* element, PCR analysis showed that all revertant alleles were the result of precise excisions with no detectable deletions. In the other, the *PBac{5HPw^+^}Lerp^A530^* insert near the 5′ end of *Lerp* was targeted for mobilization by the HoP2.1 transposase transgene. In this case, out of over 1000 adult progeny, no examples of loss of the *PBac{5HPw^+^}Lerp^A530^* insert were detected.

**Fig. 2.**
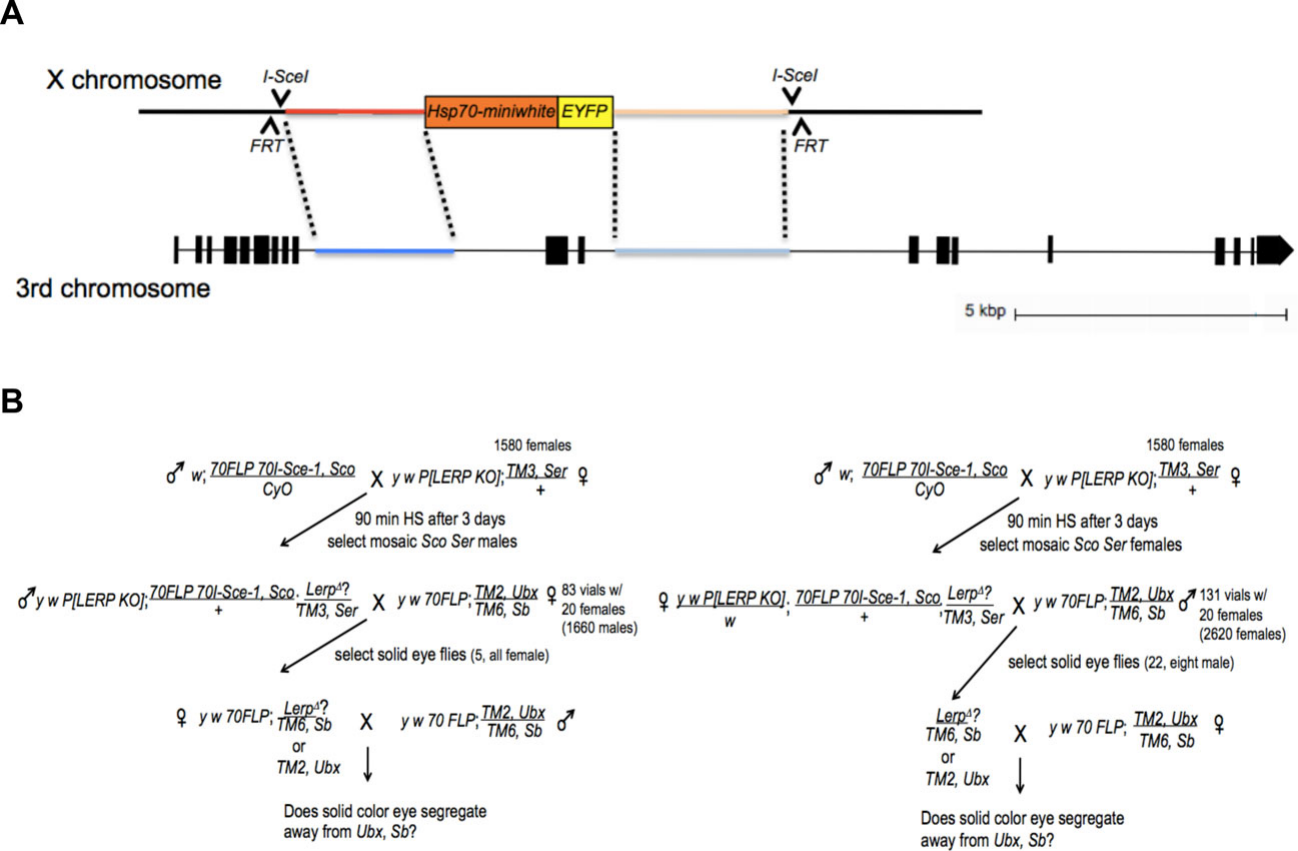
**Strategy for *Lerp* knockout.** (A) Cartoon representation of the knockout strategy. Scale is approximate. A *Lerp* knockout donor plasmid was created by cloning 2.6 kb of *Lerp* sequence upstream of the targeted exons and 2.7 kb of *Lerp* sequence downstream into multiple cloning sites of the pXH87 vector (Chen et al., 2009). This was then established as a transgene on the X chromosome by P-element-mediated germline transformation. FLP recombinase catalyzes the excision of the knockout cassette and *I-SceI* cleavage creates a linear DNA fragment from the excised circle, which then can undergo homologous recombination with the targeted genomic DNA sequence to generate a *Lerp* mutation by homologous replacement. For simplicity, the representations of the two genes to either side of *Lerp* on the chromosome are omitted. (B) Schemes to isolate *Lerp* knockout mutations generated in the male (left) and female (right) germlines.

The third strategy utilized an ends-out homologous recombination strategy based on Chen et al. (2009). A construct containing a *miniwhite* transgene, under the control of the *Hsp70Aa* promoter, and the coding sequence for enhanced yellow fluorescent protein (EYFP) was flanked by intron sequences derived from the *Lerp* locus; the *Lerp* sequences, in turn, are flanked by *FRT* sites for Flip recombinase and by target sites for the *I-Sce1* megaendonuclease (Fig. 2A). The *Lerp* knockout targeting cassette was established as a transgene on the X chromosome. We used schemes (Fig. 2B) in which the targeting cassette is excised by FLP recombinase and the subsequent DNA circle is linearized by *I-SceI*. Candidates for *Lerp* knockout by homologous recombination were selected based on retention of eye pigmentation after loss of the X-linked donor transgene, and subsequent crosses showing linkage of the donor transgene to the third chromosome. Of ∼20,000 progeny scored, three candidates were identified from mobilization in the female germline and none from mobilization in the male germline, based on mobilization of the Hsp70-*miniwhite* marker to the third chromosome. For all three candidate *Lerp* knockouts, adults homozygous for the donor transgene were identified. We were able to confirm one line, *Lerp^F6^*, in which LERP was knocked out.

### Structure of the *Lerp^F6^* allele

**Fig. 3.**
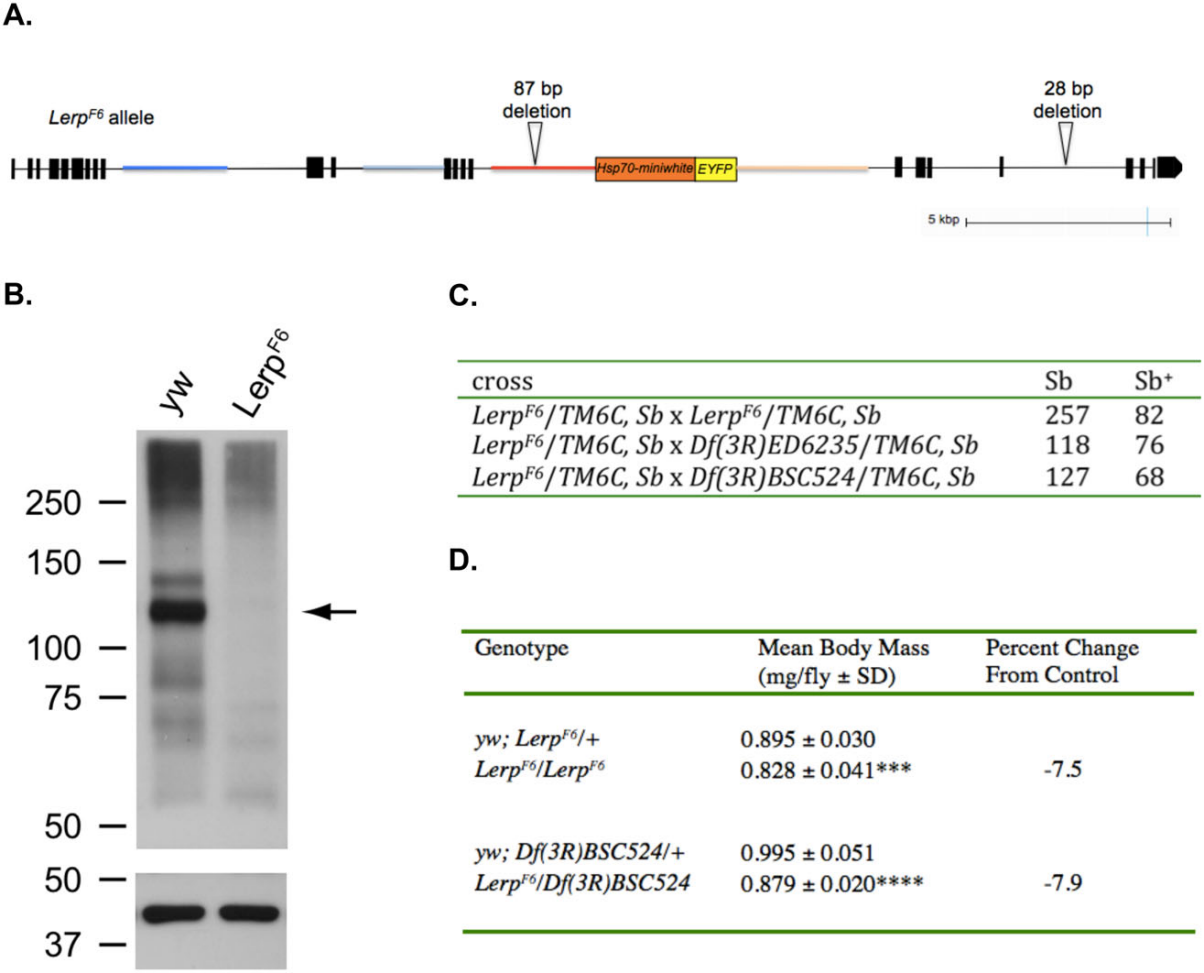
***Lerp^F6^* is a null allele of *Lerp*.** (A) Map representation of the *Lerp^F6^* allele. Scale is approximate. 87 bp and 28 bp deletions with respect to the reference sequence are indicated; these probably represent polymorphisms. (B) Western blot of total midgut protein, showing LERP protein in control (*yw*) and *Lerp^F^* extracts (top). Same blot probed with antibody to cytoplasmic actin as a loading control (bottom). (C) Crosses to test semi-lethality associated with the *Lerp^F6^* allele. Homozygous *Lerp^F6^* adults are recovered at significantly lower frequency (*P*=0.0004, Chi squared test) compared to heterozygous sibs (first line), but hemizygous *Lerp^F6^* adults appear at Mendelian frequencies compared to sibs carrying a wild-type *Lerp* allele (second and third line). (D) Body weight of *Lerp* homozygous mutants (*Lerp^F6^*/*Lerp^F6^*) and hemizygous mutants (*Lerp^F6^*/*Df(3R)BSC524*), as compared to genetic controls, *yw; Lerp^F6^/+* and *yw; Df(3R)BSC524/+*. Body weight is expressed in mg/fly. The values are means±s.d. of twelve sets of 10 male flies; *n*=120; ***P<0.001; ****P<0.0001.

To further analyze the knockout, genomic DNA sequence was obtained from *Lerp^F6^* homozygous flies. Three libraries of DNA, starting with 0.4 kb, 3 kb and 10 kb average fragment length, were sequenced to a depth of 30× using the Illumina MiSeq. The sequence analysis of these data demonstrated that the *Lerp^F6^* allele is the result of a partial internal duplication combined with an insertion that places a nearly intact copy of the donor sequence downstream of the intended target sequence. This results in duplications of the intronic sequences flanking the donor, as well as duplication of 3.5 exons from the 5′ cluster of *Lerp* exons (Fig. 3A). If the *Lerp^F6^* allele were transcribed in its entirety, the exon splice that would join the *Lerp* exons flanking the donor sequence would create a reading frame shift and in-frame stop, predicting a truncated protein missing the trans-membrane and cytoplasmic domains. Thus, the hypothetical protein product of *Lerp^F6^* would not be functional, and as a truncated peptide, would likely be unstable.

Microarray transcription profiling analysis of larval midgut tissue, where *Lerp* is very highly expressed normally (Brown et al., 2014; Dos Santos et al., 2015), indicates that *Lerp* transcripts containing the 3′ exon are detectable in mutant midgut cells at ca. 27% of wild type levels (M.H. and J.C.E., unpublished data). LERP protein expression was tested using western blot analysis in gut tissue isolated from *yw* control and *Lerp^F6^* homozygous mutant larvae (Fig. 3B). In the guts derived from *yw* control flies, LERP was detected as two bands between 100 and 150 kDa; these bands were not detected in *Lerp^F6^* mutant guts. Thus, *Lerp^F6^* mutant flies do not express detectable LERP protein.

To test for possible semi-lethality associated with the *Lerp* null mutation, we crossed *Lerp^F6^/TM6C, Sb* adults *inter se* and scored progeny. Because *TM6C, Sb* homozygotes are not viable, the expected Mendelian ratio is 2 *Sb*: 1 *Sb^+^*. The observed ratio shows a statistically significant (*P*=0.0004) deviation from the expected ratio, indicating semi-lethality associated with the *Lerp^F6^* chromosome (Fig. 3C). To test if the observed semi-lethality maps to the *Lerp^F6^* mutant allele, we tested whether semi-lethality occurs in flies hemizygous for *Lerp^F6^* and either of two independently isolated chromosome deficiencies that contain a deletion spanning the *Lerp* locus. The hemizygous crosses show a return to a 2:1 ratio (Fig. 3C), indicating that the observed semi-lethality is not due to the loss of *Lerp*. Thus, *Lerp* is not an essential gene under standard laboratory culture conditions. Although viable and fertile, both homozygous and hemizygous adult mutant flies exhibit a small but statistically significant decrease of ca. 8% (*P*<0.001 and *P*<0.0001, respectively) in body mass relative to their respective genetic controls (Fig. 3D). There is no significant difference in third instar larval weight (supplementary material Fig. S1). Decrease in adult body mass is the only morphological phenotype observed in *Lerp* null flies.

### Cellular levels of lysosomal hydrolases are reduced in *Lerp* null tissue

To determine whether the loss of LERP results in alterations in lysosomal enzyme content, we measured the activities of three lysosomal glycosidases in *Lerp^F6^* mutant and *yw* control carcasses and hemolymph. Some cell types of mice deficient in the two MPRs are defective in sorting lysosomal enzymes and as a result, most of the newly synthesized lysosomal enzymes expressed in those cells are secreted into the bloodstream (Dittmer et al., 1998). If LERP functions to sort the lysosomal enzymes in an analogous manner, we would expect to find decreased levels of these enzymes in the carcass and increased levels in the hemolymph. As shown in Table 2, the levels of β-hexosaminidase, α-mannosidase and β-glucuronidase activity were decreased by 30–40% in the carcasses of *Lerp^F6^* larvae compared to control, consistent with a role for LERP in sorting of lysosomal hydrolases to lysosomes (Table 2). However the activity of these hydrolases in the hemolymph of the *Lerp* mutant was also decreased relative to the controls. This indicates that the low level of glycosidases in the carcass is not the consequence of missorting into the hemolymph.

**Table 2.**
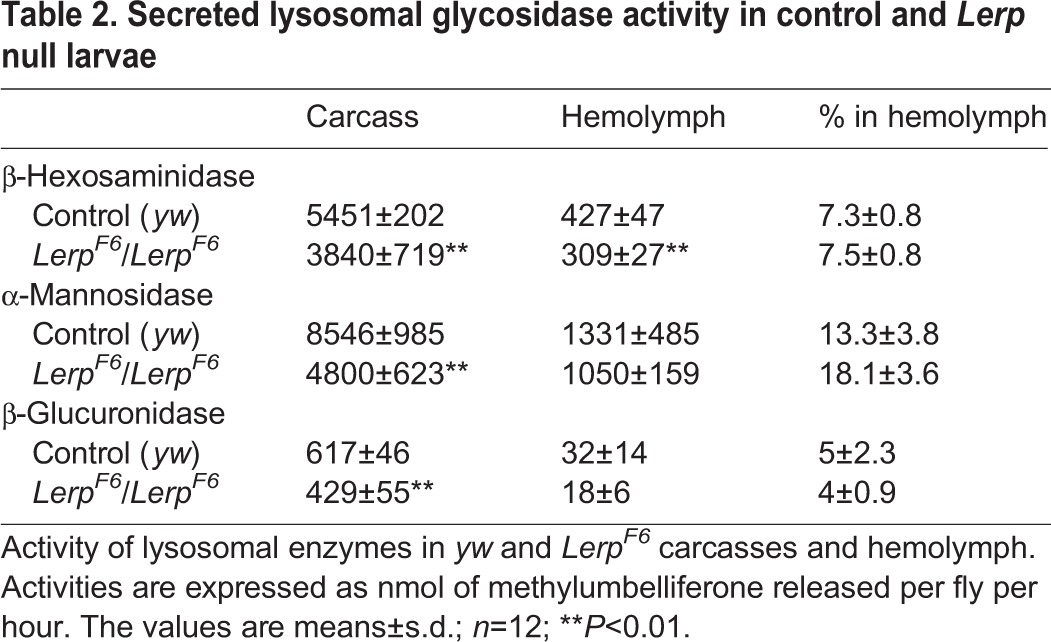
**Secreted lysosomal glycosidase activity in control and *Lerp* null larvae**

**Fig. 4.**
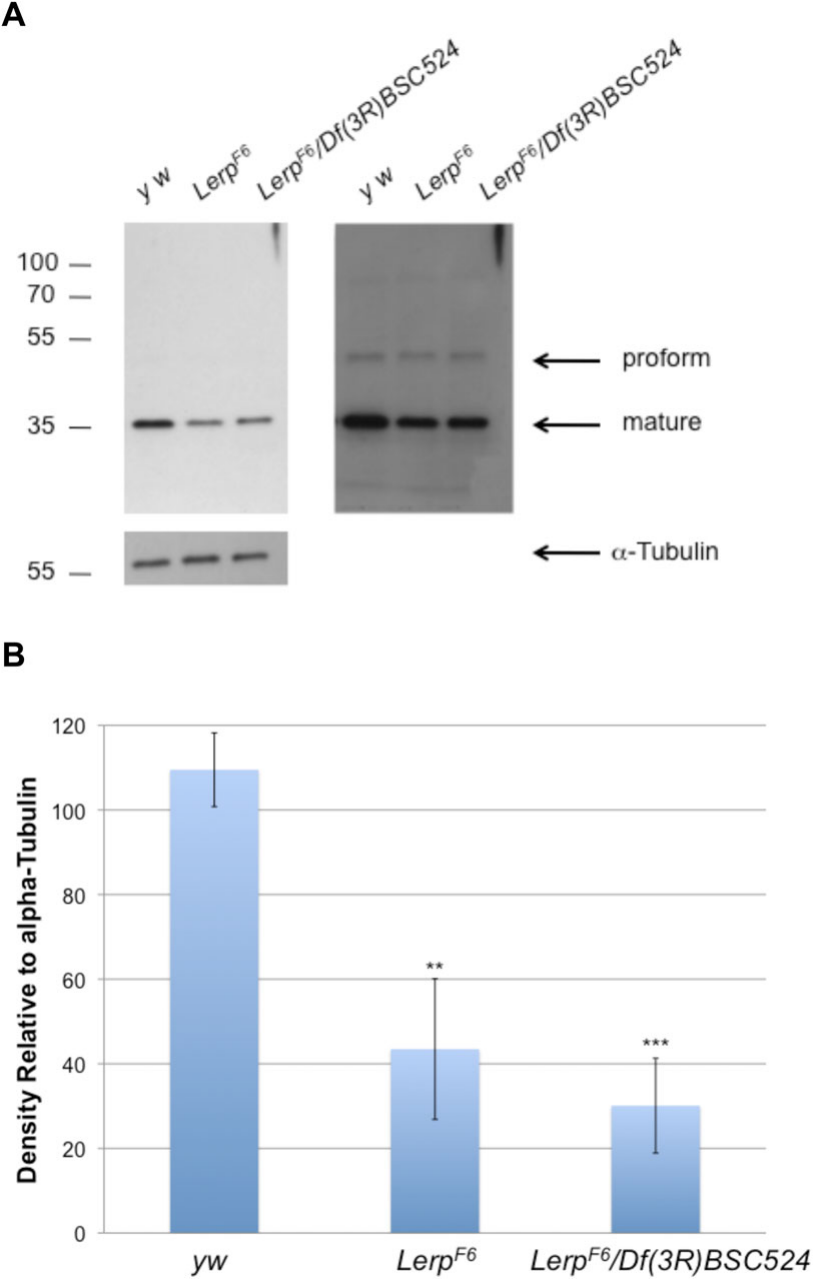
**Cathepsin L protein levels are decreased in *Lerp* mutants.** (A, left panel) Western blot (30 s exposure) showing mature CTSL protein levels (∼36 kDa) in whole third instar larval control (*yw*), homozygous *Lerp* mutant (*Lerp^F6^*), and hemizygous *Lerp* mutant (*Lerp^F6^*/*Df(3R)BSC524*) *Drosophila* samples. The unprocessed, or pro-form, of cathepsin L is not detected at the given exposure. (A, right panel) Western blot (15 min exposure) showing mature CTSL protein levels in whole third instar *yw*, *Lerp^F6^*, *Lerp^F6^*/*Df(3R)BSC524 Drosophila* samples. The unprocessed, or pro-form, of cathepsin L is more apparent at a longer exposure. Equal loading was confirmed by probing blot with α-tubulin (B) Comparison of cathepsin L protein levels by densitometric analysis. Each genotype (*n*=3 samples; 1 larvae/sample) is calculated relative to α-tubulin levels. The values are means±s.d.; **P<0.01; ***P<0.001.

We also measured the levels of the lysosomal protease cathepsin L in third instar *Lerp^F6^* homozygous, *Lerp^F6^* hemizygous, and *yw* control whole larvae by western blotting. This analysis showed a significant decrease in the level of the protease in both homozygous and hemizygous *Lerp^F6^* mutant midgut relative to the control (Fig. 4). Specifically, the levels of the mature, or lysosomal, form of cathepsin L (∼35 kDa) are decreased in mutant cells. The cellular levels of the proforms of cathepsin L (∼50 kDa) are unchanged between the mutants and the control.

The decrease in steady state levels of the lysosomal hydrolases in the LERP deficient cells could be the result of reduced synthesis. We determined the transcript levels of the unique lysosomal hydrolase gene *Cp1* (encodes cathepsin L) in mutant and control larval midguts. The values did not differ significantly when assayed by microarray analysis (M.H. and J.C.E., unpublished data). This suggests that the observed defects are not due to decreased expression of the genes encoding these enzymes.

### *Ler*p null adult flies are hypersensitive to dietary chloroquine

**Fig. 5.**
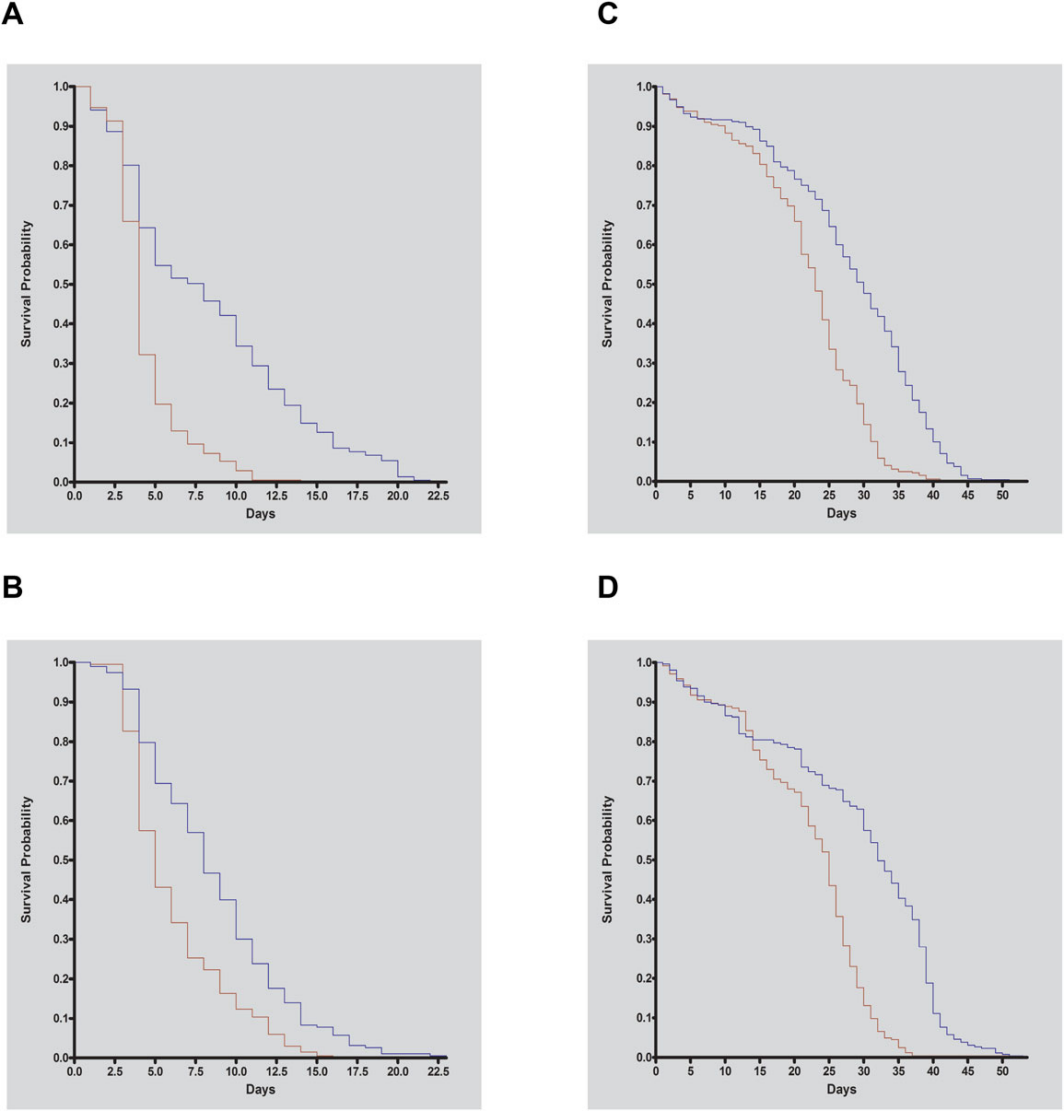
***Lerp* mutant flies are hypersensitive to dietary chloroquine and amino acid starvation.** (A,B) Blue lines represent (A) control females *yw*; *Lerp^F6^*/+; *n*=221 and median survival time eight days) and (B) control females (*yw*; *Df(3R)BSC524*/+; *n*=193 and median survival time eight days) exposed to 20 mM chloroquine. Red lines represent (A) homozygous *Lerp* mutant female (*Lerp^F6^*/*Lerp^F6^*; *n*=208 and median survival time four days) and (B) hemizygous Lerp mutant females (*Lerp^F6^*/*Df(3R)BSC524*; *n*=202 and median survival time five days) exposed to 20 mM chloroquine; *P*<0.0001. (C,D) Blue lines represent (C) control males (*yw*; *Lerp^F6^*/+; *n*=457 and median survival time 30 days) and (D) control males *yw*; *Df(3R)BSC524*/+; *n*=261 and median survival time 32 days) exposed to amino acid starvation. Red lines represent (C) homozygous *Lerp* mutant males (*Lerp^F6^*/*Lerp^F6^*; *n*=325 and median survival time 23 days) and (D) hemizygous *Lerp* mutant males (*Lerp^F6^*/*Df(3R)BSC524*; *n*=244 and median survival time 25 days) exposed to amino acid starvation; *P*<0.0001.

Exposure of *Drosophila* to 10–20 mM dietary chloroquine, a drug that raises lysosomal pH and impairs lysosome hydrolytic activity, is lethal to wild-type flies over a period of days (Luan et al., 2012). We hypothesized that if loss of LERP impairs lysosomal activity, further impairment due to chloroquine exposure would enhance the lysosomal defect and result in enhanced lethality. To test this, crosses were set up to generate homozygous and hemizygous mutant flies and the corresponding genetic controls using standard *Drosophila* food. Newly eclosed flies were then transferred to instant *Drosophila* medium reconstituted with 20 mM chloroquine. The median survival time for homozygous and hemizygous *Lerp^F6^* mutants was four and five days, respectively, compared to a median survival time for the controls of eight days (Fig. 5A,B). Thus, the survival time is significantly reduced in the *Lerp* mutants (*P*<0.0001), consistent with a role for LERP in lysosomal homeostasis.

### *Lerp* null adult flies have conditional phenotypes of autophagy defects

Autophagy is a lysosome-mediated pathway that degrades cytoplasmic material and organelles (Eskelinen and Saftig, 2009). It is activated during stress conditions, including amino acid starvation, to help cells meet the minimum nutrient requirements of starving cells (Scott et al., 2004). We reasoned that if lysosomal activity is impaired in *Lerp* null flies, lysosome-mediated pathways, including autophagy, would also be impaired. To test this, autophagy was induced by maintaining newly eclosed flies on amino acid deficient medium (Scott et al., 2004). Crosses were set up to generate homozygous and hemizygous mutant flies and corresponding genetic controls. During amino acid starvation, the median survival time for homozygous *Lerp^F6^* mutants and hemizygous *Lerp* mutants was 23 days and 25 days, respectively, compared to 30 and 32 days for the corresponding genetic controls (*P*<0.0001) (Fig. 5C,D). The reduced survival in the *Lerp* null flies is in agreement with impaired lysosome function in these flies.

**Fig. 6.**
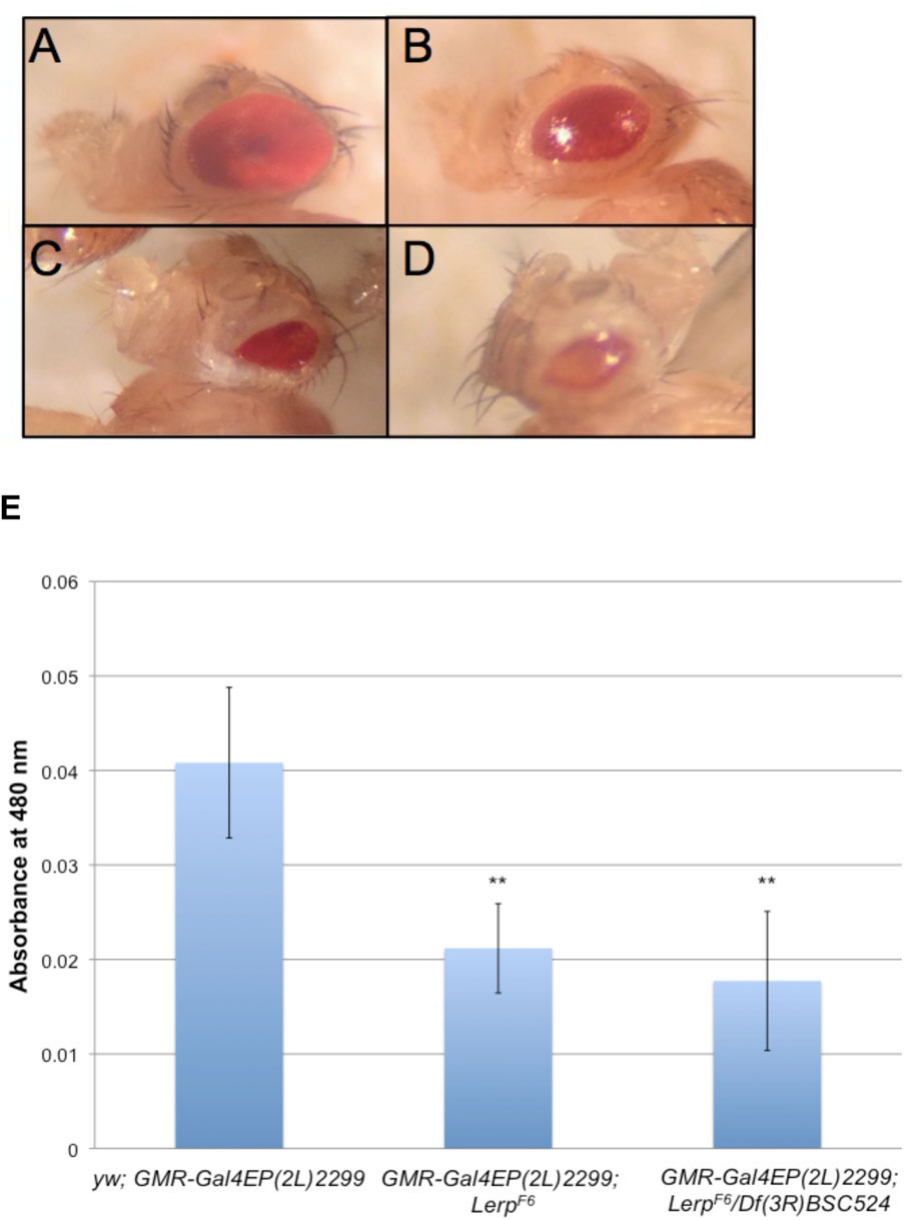
***Lerp* knockout enhances the reduced eye phenotype caused by *Bchs* overexpression.** Image depicting differences in eye size and morphology of adult flies in the presence/absence of LERP in a *Bchs* overexpression background (A) *Ore-R* (B) *yw; GMR-Gal4EP(2L)2299* (C) *GMR-Gal4EP(2L)2299; Lerp^F6^* (D) *GMR-Gal4EP(2L)2299; Lerp^F6^/Df(3R)BSC524*. (E) Amount of extracted red eye pigment was measured at 480 nm with 10 fly heads per group. The values are means±s.d.; **P<0.01.

To further test the role of *Lerp* in autophagy, we examined the interaction of *Lerp^F6^* with the autophagy-associated gene *Blue cheese* (*Bchs*). Overexpression of *Bchs* in the *Drosophila* eye causes a reduced eye phenotype, which is modified by mutations in genes thought to be involved in autophagy (Lim and Kraut, 2009; Simonsen et al., 2007). We tested the effects of loss of *Lerp* expression on the *Bchs* overexpression phenotype. The differences of eye size between *Bchs* overexpression in control, homozygous and hemizygous mutant flies was quantified by measuring the amount of red eye pigment in each genotype as an index of total eye volume. *Lerp* knockout in a *Bchs* overexpressing background enhances the reduced eye phenotype, directly or indirectly implicating LERP in autophagy (Fig. 6).

## DISCUSSION

RNAi knockdown of *Lerp* in *Drosophila* S2 cultured cells resulted in no significant reduction in cellular levels of five lysosomal glycosidases, nor in cellular levels of the lysosomal protease cathepsin L. This is consistent with a previous report, also based on RNAi knockdown in S2 cells, suggesting that LERP is not a universal sorting receptor for lysosomal proteins in flies (Kowalewski-Nimmerfall et al., 2014). However, it should be noted that *Lerp* expression is normally low to moderate in S2 cells (Cherbas et al., 2011; Dos Santos et al., 2015), so a strong but incomplete knockdown of *Lerp* may not result in measurable sorting defects.

Our successful generation of a *Lerp* knockout mutant in *Drosophila* has allowed us to test the role of this transmembrane protein in development and in lysosome formation and function in an intact organism. We find that LERP is not essential for development or fertility under standard laboratory conditions, although growth is mildly impaired. The external appearance of *Lerp^F6^* adults is normal. In particular, the compound eyes of newly eclosed flies are wild-type in appearance. This is notable in that Kametaka et al. (2010) reported that knockdowns of the σ, γ and µ subunits of the adaptor protein AP-1 in the developing eye results in a rough eye phenotype in adults. While AP-1 is believed to contribute to LERP-dependent sorting, the observation that *Lerp* null adults have normal eyes shows that the reported AP-1 knockdown phenotypes are LERP-independent.

Since LERP is an ortholog of the CI-MPR and has been reported to partially rescue sorting of lysosomal hydrolases in MPR-deficient mammalian cells (Dennes et al., 2005), we were especially interested in determining whether the LERP mutant flies exhibited defects in lysosome biogenesis and function. Loss of the MPRs in mice results in a lysosomal storage phenotype in many tissues and increased levels of lysosomal enzymes in the serum (Dittmer et al., 1998, 1999). Loss of LERP in flies, however, results in only mild phenotypes under standard lab conditions. A moderate reduction in the level of mature cathepsin L was observed in the midgut. In addition, by assaying carcass tissue freed of hemolymph, we found that the LERP mutant had a 30–40% decrease in the level of several lysosomal glycosidases relative to wild-type flies. However, the levels of these glycosidases were not increased in the hemolymph, indicating that the enzymes were not missorted into the hemolymph. We cannot exclude the possibility that the hydrolases are being missorted elsewhere. *Drosophila* larval midgut and malpighian tubules, which express high levels of LERP, are highly polarized cells (Tepass et al., 2001). Thus, the hydrolases might be missorted apically into the lumen of the gut and subsequently excreted. Regardless of the explanation for the decreased levels of lysosomal hydrolases in the LERP mutant, a key finding of this study is that *Lerp* mutant cells retain 60–70% of wild-type levels of α-mannosidase, β-glucoronidase, and β-hexosaminidase, and possibly other enzymes. These findings establish that acid hydrolases are trafficked to lysosomes in a LERP-independent manner.

Since cellular lysosomal enzyme levels are reduced in *Lerp* mutants, we considered the possibility that lysosome-dependent processes, such as autophagy, might be impaired. That this is the case is supported by the observation that *Lerp* mutant flies are hypersensitive to amino acid starvation, consistent with inefficient autophagy. Further evidence of cellular lysosome impairment in *Lerp* null flies is indicated by the hypersensitivity of *Lerp* mutants to dietary chloroquine and the enhancement of the reduced eye phenotype in *Bchs* overexpressing flies.

*Lerp* is the only recognizable MPR ortholog in *Drosophila*. Why has it been conserved evolutionarily if it is not essential? It is likely that laboratory culture conditions don't adequately recapitulate the selective pressures experienced by wild flies. In particular, transient starvation is frequently experienced by animals in nature, so the hypersensitivity of *Lerp* null adults to amino acid starvation represents a conditional phenotype that could underlie an essential function for *Lerp.*

The mechanism by which LERP influences lysosomal enzyme levels remains open. It should be noted that the mammalian CI-MPR binds multiple ligands in addition to lysosomal hydrolases (Ghosh et al., 2003). These include IGF-II, latent TGF-B1, retinoic acid and others. Since direct binding of lysosomal hydrolases to LERP has not been documented as yet, the possibility that LERP has an indirect effect on lysosome biogenesis cannot be excluded at this point. Future studies should first be aimed at defining the ligands for LERP. Once ligands are identified, biochemical and cell biology approaches can be used to determine the physiologic role of LERP.

## MATERIALS AND METHODS

### LERP knockdown in *Drosophila* S2 cells

S2 cells were maintained at room temperature in Express Five SFM culture medium (Life Technologies) supplemented with 2 mM L-glutamine (Cellgro; Manassas, VA, USA), 100 U/ml penicillin and 100 μg/ml streptomycin (Life Technologies). To knockdown LERP, two dsRNAs (∼670 and ∼800 nucleotide fragments) targeting different regions of the LERP mRNA were generated. First, total RNA was isolated from *Drosophila* S2 cells using TRIzol Reagent (Life Technologies) and cDNA was synthesized with the SuperScript II RT kit (Life Technologies) according to the manufacturer's protocols. PCR was performed with gene-specific primers flanked by the T7 RNA polymerase promoter sequence at the 5′-ends, as described in Rogers and Rogers (2008). The following primers were used: LERP1-forward: 5′ TAA TAC GAC TCA CTA TAG GCC TGC AGG TGA CAA AAT GCG 3′ and reverse: 5′ TAA TAC GAC TCA CTA TAG GCT GCA ACT ATT GGA TTG TAG ACC CTC 3′, LERP2-forward: 5′ TAA TAC GAC TCA CTA TAG GCA GCT CGC ACT TTG CTT AAG GAT G 3′ and reverse: 5′ TAA TAC GAC TCA CTA TAG GCG TTG AGA GCT CCG AGG TGT TG 3′ and Rho1 (control dsRNA) forward: 5′ TAA TAC GAC TCA CTA TAG GTT TGT TTT GTG TTT AGT TCG GC 3′ and reverse: 5′ TAA TAC GAC TCA CTA TAG GAT CAA GAA CAA CCA GAA CAT CG 3′. *In vitro* transcription was performed with the MEGAscript RNAi kit (Ambion) as instructed by the manufacturer.

In RNAi experiments, 2×10^6^ S2 cells were transfected with 2 μg dsRNA using Lipofectamine Plus (Life Technologies) and analyzed 5 days later. Mock-treated and mock-depleted cells were transfected without the addition of dsRNA or with Rho1 dsRNA, respectively. The level of knockdown relative to GAPDH (primers Cat. #330001 PPD03944A, Qiagen) was determined by quantitative RT-PCR using SYBR green master mix (SA Biosciences) and 10 μM primers to LERP (Cat. #330001 PPD10274A, Qiagen). To evaluate the secretion of lysosomal enzymes into the culture medium, the cells were washed with PBS and incubated with fresh culture medium approximately 16 h before the analysis. The S2 cells were lysed in 1% Triton X-100/PBS containing a protease inhibitor cocktail (Complete, Roche) and the activities of β-hexosaminidase, β-glucuronidase, α-mannosidase, β-mannosidase and β-galactosidase were determined as described below.

Pulse-chase labeling experiments were performed with S2 cells that were treated with LERP RNAi for 5 days or mock-treated, as described in van Meel et al. (2014) with minor modifications. The pulse labeling was performed in methionine/cysteine-free, serum-free DMEM supplemented with 18 mM L-glutamine for 20 min at room temperature. Cathepsin L was immunoprecipitated after a 4 h chase with the antibody (MAB22591) from R&D Systems, Inc.

For western blot analysis, 15–20 μg of cell lysate was separated by SDS-PAGE on an 8% (in the case of LERP) or 12% (cathepsin L) Tris-glycine gel and subsequently transferred to 0.2 μm nitrocellulose membranes (Amersham Protran, GE Healthcare U.K. Limited). LERP was detected with the antiserum described below at dilution 1:1000–1:2000 and cathepsin L with an antibody from R&D Systems, Inc (MAB22591) at dilution 1:1000. Secondary antibodies used were donkey anti-rabbit or sheep anti-mouse IgG Horseradish peroxidase linked whole antibody (GE Healthcare U.K. Limited), respectively, at dilution 1:2000.

### Production of recombinant LERP

For production of antibodies to LERP, the LERP cDNA encoding amino acids 1–816 encompassing the luminal domain of the protein was cloned into the baculovirus shuttle vector, pFastBac1, with the Flag epitope sequence appended to the 3′ end of the cDNA. Baculoviral bacmid DNA isolated from DH10Bac cells was transfected into *Spodoptera frugiperda* (SF9) insect cells adapted for growth in serum-free media (Life Technologies). Viral particles in the media were amplified for two rounds and subsequently used to infect SF9 cells for protein production. Since the LERP construct used here lacked the C-terminal transmembrane and cytoplasmic domains, the protein was secreted into the serum-free media.

The soluble LERP secreted into the media was purified on a Flag affinity column (Sigma), concentrated and used to generate antibodies as follows: approximately 100 µg of purified soluble LERP diluted in sterile saline was combined with 0.5 ml of complete Freund's adjuvant and injected subcutaneously into 2 rabbits. Two weeks following the first injection, booster shots of 50 µg were administered in incomplete Freund's adjuvant and repeated again after another two weeks. Rabbits were bled 6 weeks after the initial injection to check for antibody production and a terminal bleed was performed at 6 months.

### *Drosophila* stocks

The *w^1118^; Mi{ET1}Lerp^MB05321^*, *y^1^ w^1118^; PBac{5HPw^+^}Lerp^A530^*, *w^1118^; Df(3R)ED6235*, /*TM6C, cu^1^ Sb^1^*, *w^1118^*; *Df(3R)BSC524*/*TM6C*, *Sb^1^* y^1^w*; *CyO, H{PΔ2-3}HoP2.1/Bc^1^*, *y w*; *[70FLP][70I-SceI], Sco*/*CyO*, *w*; *[70FLP]; TM3, Ubx/TM6, Sb^1^*, and *Df(3R)BSC524*/*T(2,3)CySerGFP* stocks were obtained from the Bloomington *Drosophila* Stock Center. The *GMRGal4EP(2L)2299* stock was obtained from Dr K. Finley, San Diego State University. All crosses were maintained on standard cornmeal-agar-molasses-yeast food at 25°C unless otherwise indicated.

### Strategy for *Lerp* targeted knockout

The overall approach for targeted knockout is described in Chen et al. (2009) and the strategy design is cartooned in Fig. 2A. The donor cassette was flanked by *Lerp* genomic sequences 3R:22,684,293-3R:22,686,400 and 3R:22,677,487-3R:22,680,473. Ca. 2.6 kb upstream of the Lerp exons targeted for knockout (using primers forward: 5′ CGGCCTCGAG TGGCTCTCAGGACCATAATC 3′ , reverse: 5′ CCAGCTAGCCAAAAAAAGCGAGGCCTGCGAAAAG 3′) was amplified from genomic DNA and cloned into the pXH87 vector with *Xho*I and *Nhe*I sites. Ca. 2.7 kb downstream of the Lerp exons targeted for knockout (using primers forward: 5′ CGACCGGTCTCGCAACCAGATTTCACCCAGGAC 3′, reverse: 5′GCCGGTACCCAGATGAGCGGGGATGAGAGGAG 3′) were amplified from genomic DNA and cloned into the pXH87 vector with *Age*I and *Kpn*I sites. Plasmid DNA was sent to BestGene Inc. (Chino Hills, CA, USA) to generate transgenic flies. Transgenic flies were selected based on eye pigmentation conferred by the *Hsp70-miniwhite* gene in the donor cassette.

### *Lerp^F6^* genome sequencing

Genomic DNA was extracted from flies by homogenization in 100 mM Tris-HCl (pH 7.5)/100 mM EDTA/100 mM NaCl/0.5% SDS, followed by phenol extraction, chloroform extraction and ethanol precipitation. The genomic DNA was quantified using qubit fluorometry (Life Technologies) and 4 μg was used as input to the Illumina Nextera XT library preparation protocol. Three libraries were prepared: 350 bp, 4 kbp, and 9 kbp. Tagmentation of gDNA, and PCR amplification of tagged DNA were performed as per manufacturer's (Illumina) instructions. For the 350 bp library PCR clean up and library normalization steps were performed per Illumina protocol. However, for the longer length libraries PCR Clean-Up and Library Normalization steps were omitted and size selection was instead performed by running balanced and pooled samples in a 0.6% agarose gel. Gel fractions corresponding to 3–5 kb, 8–10 kb were removed and purified using Zymoclean large fragment DNA recovery kit. The size selected DNA was circularized and remaining linear fragments were eliminated using exonuclease. The circularized fragments were fragmented using Covaris sonicator. AMPure XP beads (Agilent Technologies) were used to purify the DNA and Illumina Truseq adapters were ligated to the ends of the DNA fragments. The fragments were captured on beads and emulsion PCR performed per Illumina's protocol. 4 nM of beads were sequenced using paired-end 250 nucleotide reads on Illumina MiSeq.

For assembly and annotation, reads from all three libraries were assembled using wild-type *Drosophila* genome (Celniker et al., 2002) as reference in Illumina BaseSpace. The analysis of the disrupted *Lerp* locus was performed manually using the UCSC genome browser and custom scripts written for mapping all the reads containing at least some from eYFP and *Lerp* sequence and aligning that portion of the read to the locus.

### Measuring *Drosophila* body mass

*Lerp^F6^* virgin females were crossed to *Lerp^F6^* males, *yw* males, and deficiency males (*Df(3R)BSC524*/*T(2,3)CySerGFP*), and *yw* virgin females were crossed to deficiency males to generate homozygous and hemizygous knockout and control flies. Immediately following eclosion, males were collected and aged for 24 h on standard *Drosophila* media. Measurements were recorded using 10 flies in a 1.5 ml Eppendorf tube per reading. Eppendorf tubes were pre-weighed and fly mass was determined by subtracting mass of the Eppendorf tube alone from the total mass of flies plus the tube. *Lerp^F6^*, *yw*, and *Lerp^F6^/Df(3R)BSC524* male and female third instar larvae were grown on instant *Drosophila* media (Carolina Biological Supply Company) reconstituted with a 0.05% Bromophenol Blue water solution (Sigma Aldrich) and staged 6–12 h prior to pupariation (Andres and Thummal, 1994). Measurements were recorded using 5 larvae in a 1.5 ml Eppendorf tube per reading. Eppendorf tubes were pre-weighed and larval mass was determined by subtracting mass of the Eppendorf tube alone from the total mass of flies plus the tube.

### Chloroquine survival curves

*Lerp^F6^* virgin females were crossed to *yw* males, deficiency males (*Df(3R)BSC524*/*T(2,3)CySerGFP*), and *yw* virgin females were crossed to deficiency males to generate homozygous and hemizygous knockout and control flies. Flies were raised on normal fly food until pupation, and then transferred onto chloroquine-containing media, which consists of 2 g instant *Drosophila* media (Carolina Biological Supply Company) reconstituted with 6 ml of 20 mM chloroquine (Sigma-Aldrich), 0.3% Proprionic acid, and 0.3% Tegosept. The number of surviving flies was recorded daily.

### Starvation test

Flies were raised on normal fly food until pupation, and then transferred to amino acid-deficient food (3% agar, 5% sucrose, 0.3% methylparaben and 0.3% proprionic acid in PBS). Adult males were collected within 6 h of eclosion and transferred to fresh amino acid deprived food. The number of surviving flies was recorded daily.

### Lysosomal enzyme assays

The activities of β-hexosaminidase, α-mannosidase and β-glucuronidase were determined in carcasses and hemolymph using 1 mM 4-methylumbelliferyl-conjugated specific substrates (Sigma) in 50 mM sodium citrate buffer containing 0.5% Triton X-100 (pH 4.6) as previously described (Lee et al., 2007). The hemolymph was collected from larvae by the following method: 100 µl of Ringers solution was placed in a glass well chilled on ice. For each of ten consecutive larvae of each genotype, a small tear was made in the cuticle to release hemolymph into the Ringers solution. After accumulating hemolymph from 10 larvae, the well contents were placed in a microfuge tube, centrifuged at top speed for 10 min at 4°C, and the cell-free supernatant collected for assay. For each genotype, three drained carcasses were pooled and homogenized in 500 μl 1% Triton X-100/PBS containing a protease inhibitor cocktail (Complete, Roche). 10 μl of the clarified lysate or 5 μl of the hemolymph was used in each reaction. All samples were assayed in duplicate and in total 12 sets of carcasses/hemolymph of *yw* and homozygous *Lerp^F6^* larvae were assayed in four independent experiments.

### LERP western blotting

Two midguts of wild-type or *Lerp^F6^* homozygous third instar larvae were pooled and lysed in 200 μl 1% Triton X-100/PBS containing a protease inhibitor cocktail (Complete, Roche). Approximately 1/10th of the clarified lysate was subjected to SDS-PAGE using a NuPAGE 4–12% Bis-Tris gel and NuPAGE Mops SDS running buffer (Life Technologies) and the proteins were transferred to a polyvinylidine fluoride membrane (Millipore). LERP was detected with a rabbit antibody generated to a soluble form of the protein (lacking amino acids 817-886). Actin was detected with a rabbit-anti-actin antibody from Sigma (A2066).

### Cathepsin L western blotting (tissue samples)

Third instar wandering larvae were staged and single larvae were lysed in 250 μl 1% Triton X-100/PBS containing a protease inhibitor cocktail (Complete, Roche). A standard Lowry protein assay was performed and ∼10 μg of the clarified lysate was subjected to SDS-PAGE using a 10% Bis-Tris gel, and the proteins were transferred to a polyvinylidine fluoride membrane (Millipore). Tubulin antibody (1:3000) was purchased from Sigma (T9026); Mouse anti-insect cathepsin L antibody (1:4000) was purchased from R&D Systems, Inc. (MAB22591). HRP-conjugated goat anti-mouse antibodies were purchased from Millipore.

### Analysis of *Bchs* overexpression eye phenotype

*Lerp^F6^* homozygous and hemizygous mutants were generated in a *GMRGal4EP(2L)2299* background*.* Control flies were generated by crossing *yw* virgins with *GMRGal4EP(2L)2299* males. Sons were collected and aged for three days before dissection. For each replicate, 10 fly heads were cut between eyes and placed in 1 ml acidified ethanol (pH 2) for 24 h. Absorbance measurements on five replicates were taken at wavelength 480 nm.
